# A Simple Procedure for the Evaluation of Bone Vitality by Staining with a Tetrazolium Salt

**DOI:** 10.3390/ijms18081646

**Published:** 2017-07-28

**Authors:** René Schiffner, Juliane Reiche, Steffen Brodt, Olaf Brinkmann, Matthias Bungartz, Georg Matziolis, Martin Schmidt

**Affiliations:** 1 Orthopaedic Department, Jena University Hospital-Friedrich Schiller University, Campus Eisenberg, Klosterlausnitzer Str. 81, 07607 Eisenberg, Germany; S.Brodt@krankenhaus-eisenberg.de (S.B.); O.Brinkmann@krankenhaus-eisenberg.de (O.B.); M.Bungartz@krankenhaus-eisenberg.de (M.B.); G.Matziolis@krankenhaus-eisenberg.de (G.M.); 2 Institute for Biochemistry II, Jena University Hospital-Friedrich Schiller University, Nonnenplan 4, 07743 Jena, Germany; Juliane.Reiche@med.uni-jena.de (J.R.); Martin.Schmidt@med.uni-jena.de (M.S.)

**Keywords:** TTC, TTC staining, osteonecrosis, vital staining, bone vitality

## Abstract

Presently, no intra-operative method for a direct assessment of bone vitality exists. Therefore, we set out to test the applicability of tetrazolium-based staining on bone samples. The explanted femoral heads of 37 patients were used to obtain either cancellous bone fragments or bone slices. Samples were stained with 2,3,5-triphenyl-2H-tetrazolium chloride (TTC) or 3-(4,5-dimethylthiazol-2-yl)-2,5-diphenyltetrazolium bromide (thiazolyl blue, MTT) at different times (one to twelve hours) after explantation. Staining was quantified either spectrophotometrically after extraction of the dyes or by densitometric image analysis. TTC-staining of cancellous bone fragments and bone slices, respectively, indicated the detectability of vital cells in both types of samples in a window of up to six hours after explantation. Staining intensity at later time-points was indistinguishable from the staining of untreated samples or sodium azide treated samples, which represent dead cells. In contrast, MTT-staining of bone slices revealed intense unspecific staining, which obscured the evaluation of the vitality of the samples. The lack of a detectable increase of colour intensity in TTC-stained bone samples, which were treated more than six hours after explantation, corresponds to reduced fracture healing. The described simple procedure could provide a basis for an intraoperative decision by the orthopaedic surgeon.

## 1. Introduction

Macroscopic-histological examination via 2,3,5-triphenyl-2H-tetrazolium chloride (TTC) is an established method for the determination of structural damages in organs. Hitherto, this staining method has been used on tissue samples of the brain and the heart, to promptly assess the vitality of the respective tissue after organ removal. Thus, induced cerebral strokes and cardiac heart infarctions, and their respective extent, can be assessed [1,2].

The conversion of the dye’s precursor to a visible dye is hindered in damaged tissue because of a lack of mitochondrial membrane potential (concomitant with shortage of adenosine triphosphate (ATP)) in ischemic areas. This mechanism allows a discrimination of damaged or dead tissue (remains unstained) and healthy tissue (reddening) through TTC staining, and furthermore enables a quantitative comparison of the formerly named (dead and healthy, respectively) tissues. The results are rated as a reliable parameter for an early visualization of area-specific under-perfusion [1,2,3].

In this study, we tried to transfer the TTC staining method on bone tissue, specifically on human femoral heads. This could allow an assessment whether an intra-operatively removed bone tissue appears macro-histologically vital. Consequently, this might facilitate a direct intraoperative decision concerning the further therapeutic procedure, for example, whether to use a bone-conserving (revision prosthesis) or a bone-replacing (femur replacement, mega prosthesis system) approach. Furthermore, the procedure could potentially provide an indication on how long a femoral head remains viable if deprived from vascular supply. This information would then allow conclusions on how long joint preserving operations, for example in the case of dislocated medial femoral neck fractures, are still a viable option or from what time-point onwards a joint-replacing procedure should be employed.

## 2. Results

**Figure 1:**
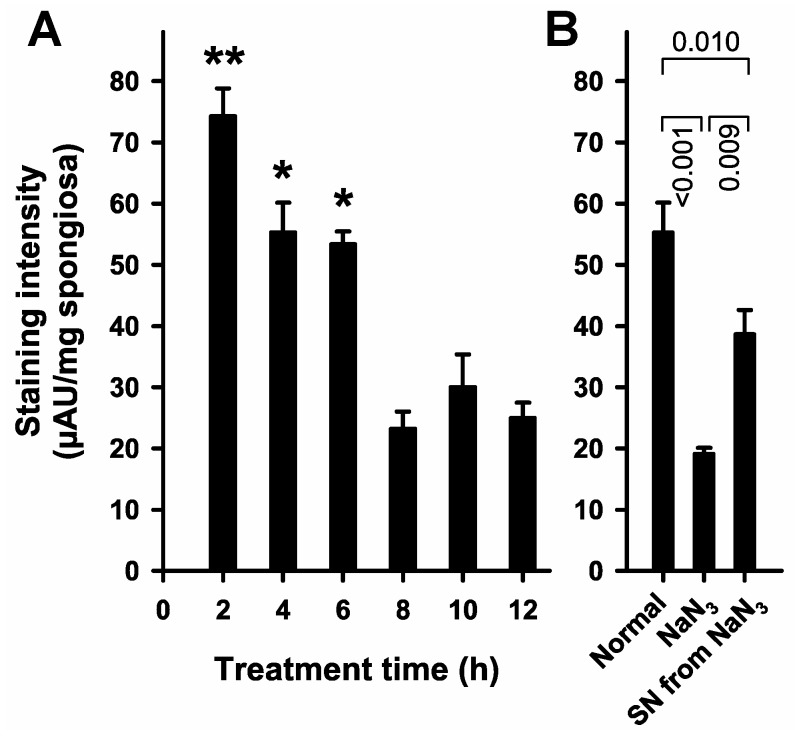
Vital staining of cancellous bone fragments with TTC. Cancellous bone fragments from femoral heads were stained with TTC as described in “Materials and Methods” Treatment time is equivalent to the time after the start of the experiments, when the staining solution was applied. (**A**) Staining intensity depends on treatment time. ANOVA (*p* < 0.001) and Holm–Sidak test (** *p* < 0.001; * *p* < 0.02, versus all times not indicated by an asterisk) revealed a significantly higher staining intensity during the first 6 h; (**B**) TTC-staining depends on vital cells. Cancellous bone fragments were stained according to the normal protocol (Normal), or after pre-treatment with NaN_3_ to determine background staining (NaN_3_). The supernatant of NaN_3_-treated cancellous bone fragments is still able to stain untreated fragments (SN from NaN_3_). ANOVA (*p* < 0.001) and Holm–Sidak test (*p*-values are given in the figure).

We used bone fragments from the cancellous bone of femoral heads to test our hypothesis that TTC-staining might be useful to detect vital cells in human bone. Maximal staining intensity was measured when staining was performed two hours upon initiation of the experiments (rewarming of the cancellous bone fragments, Figure 1A). A significant loss of staining intensity was observed when staining was performed more than six hours after start of the time-course experiments. To elucidate the level of background signal derived from non-vital cells, cancellous bone fragments were treated with the respiratory chain toxin NaN_3_ before the TTC stain was applied (Figure 1B). Treatment with NaN_3_ resulted in reduced signal intensity, comparable to signals obtained from bone fragments stained 8–12 h upon initiation of experiment. To ensure that NaN_3_ itself does not interfere with the dye, the staining solution from NaN_3_-treated cells was used to stain non-treated cells (Figure 1B). This control experiment revealed that treatment with NaN_3_ did not prevent the staining reaction itself, albeit reducing the staining intensity.

**Figure 2:**
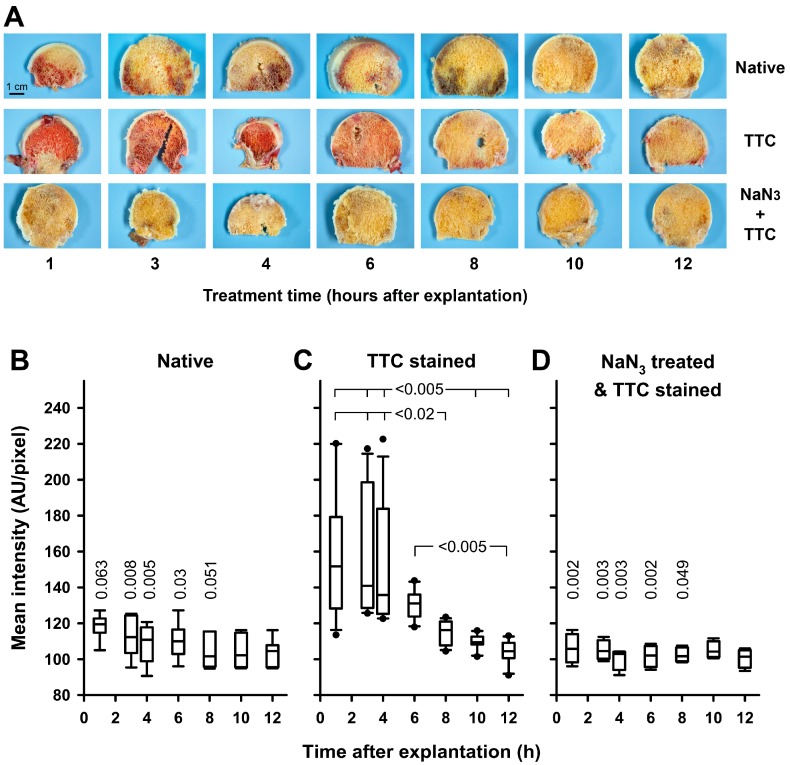
Vital staining of bone slices with TTC. Bone slices of femoral heads were stained with TTC as described in “Materials and Methods”. Treatment time is equivalent to the time after explantation, when the staining solution was applied. (**A**) Representative slices from the untreated group (Native), from the TTC-stained group (TTC) and of the group wherein TTC-staining was performed after pre-treatment with NaN_3_ (NaN_3_ + TTC) are shown, respectively. The staining intensity of bone slices was quantified by densitometric analysis with the FIJI-software as described in “Materials and Methods”; (**B**) Staining intensity of untreated slices (Native) does not depend on treatment time, *n* = 6 per time-point; (**C**) TTC-staining intensity decreases over time after explantation, *n* = 10–12 per time-point. KWAR (*p* < 0.001) with Dunn’s all pairwise multiple comparison (*p*-values are given in the figure); (**D**) Staining intensity of slices stained with TTC after pre-treatment with NaN_3_ does not depend on treatment time, *n* = 4 per time-point. Comparison of different treatments at individual time-points reveals significant differences in staining intensity for 1 h (KWAR, *p* = 0.001), 3 h (KWAR, *p* < 0.001), 4 h (KWAR, *p* < 0.001), 6 h (KWAR, *p* = 0.001), and 8 h (KWAR, *p* = 0.012), respectively. Differences to TTC-stained slices were identified with Dunn’s all pairwise multiple comparisons (respective *p*-values are given in the panels **B** and **D**).

To test whether TCC-staining might be useful to determine the vitality of cells in human bones in a setting needing no extraction of the dye, we used bone slices prepared from femoral heads. The slices were randomly assigned to three different treatment groups and seven time groups after explantation for staining. Untreated slices exhibited variable but moderate coloration from remaining blood (Figure 2A, Native). A much stronger and readily detectable colour formation was observed in TTC stained slices that were treated up to six hours after explantation (Figure 2A, TTC). In contrast, staining was completely blocked in slices that were pre-treated with NaN_3_ to kill the cells before staining (Figure 2A, NaN_3_ + TTC).

Quantitation of the red colour intensity from all images revealed no time-dependent differences in untreated slices (Figure 2B). However, the TTC-staining identified a significant proportion of vital cells in bone slices up to six hours after explantation, but not beyond this time-point (Figure 2C). In addition, NaN_3_-treatment prevented the staining of bone slices (Figure 2D).

**Figure 3:**
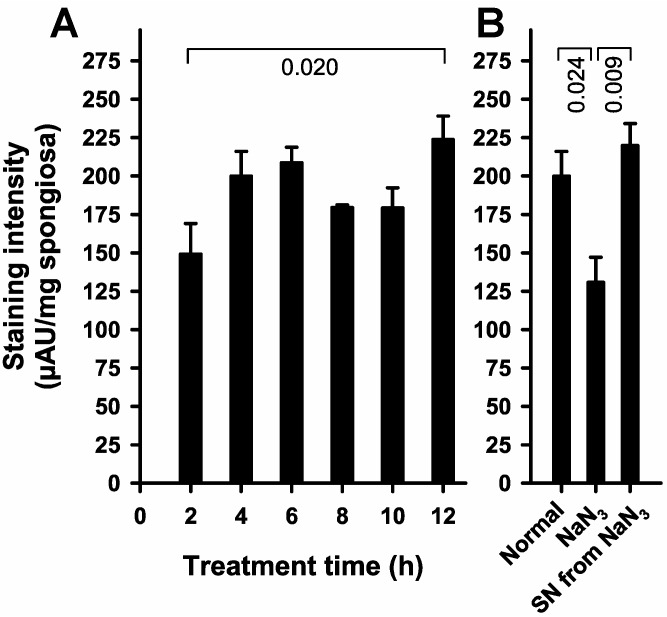
Vital staining of cancellous bone fragments with MTT. Cancellous bone fragments of femoral heads were stained with MTT as described in “Materials and methods”. Treatment time is equivalent to the time after the start of the experiments, when the staining solution was applied. (**A**) Time-dependence of staining indicates that staining is not related to the vitality of cancellous bone fragments. ANOVA (*p* < 0.02) and Holm–Sidak test (*p*-value is given in the figure); (**B**) MTT-staining partially depends on vital cells. Cancellous bone fragments were stained according to the normal protocol (Normal), or after pre-treatment with NaN_3_ to determine background staining (NaN_3_). The supernatant of NaN_3_-treated cancellous bone fragments is still able to stain untreated fragments (SN from NaN_3_). ANOVA (*p* < 0.007) and Holm-Sidak test (*p*-values are given in the figure).

To achieve faster staining and a better contrast in relation to remaining blood, the properties of the related MTT-stain seemed to be promising. Therefore, another series of cancellous bone fragments were stained with MTT (Figure 3). Bone fragments treated under all conditions, including NaN_3_ pre-treatment, exhibit intense staining, though, indicating strong unspecific staining.

**Figure 4:**
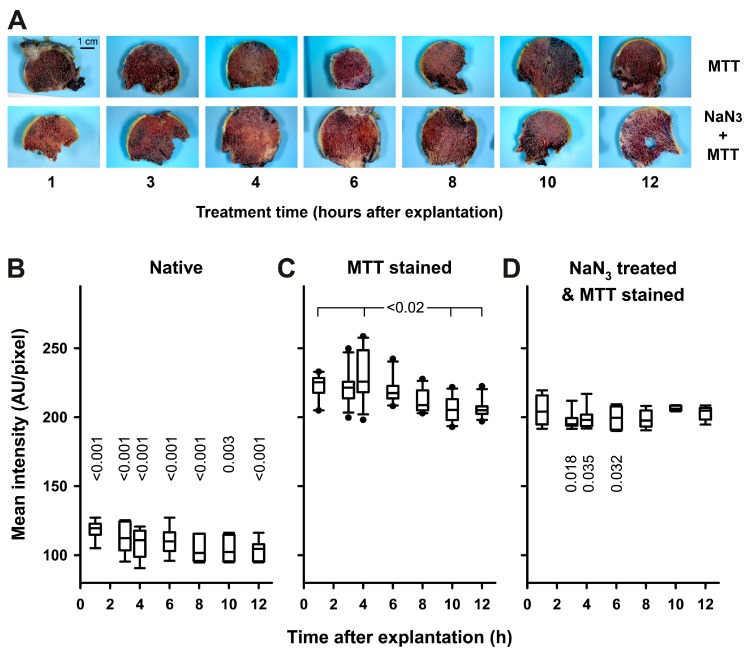
Vital staining of bone slices with MTT. Bone slices of femoral heads were stained with MTT as described in “Materials and Methods”. Treatment time is equivalent to the time after explantation, when the staining solution was applied. (**A**) Representative slices from the MTT-stained group (MTT) and from the group wherein MTT-staining was performed after pre-treatment with NaN_3_ (NaN_3_ + MTT) are shown. For comparison with untreated slices, see Figure 2. The staining intensity of bone slices was quantified by densitometric analysis with the FIJI-software as described in “Materials and Methods”; (**B**) Staining intensity of untreated slices (Native) does not depend on treatment time, *n* = 6 per time-point (using the same data-set as in Figure 2B); (**C**) MTT-staining intensity at early time-points is higher than at late time-points after explantation, *n* = 10–12 per time-point. KWAR (*p* < 0.001) with Dunn’s all pairwise multiple comparison (*p*-values are given in the figure); (**D**) Staining intensity of slices stained with MTT after pre-treatment with NaN_3_ does not depend on treatment time, *n* = 8 per time-point. Comparison of different treatments at individual time-points reveals significant differences in staining intensity for all time-points (KWAR, *p* < 0.001). Differences to MTT stained slices were identified with Dunn’s all pairwise multiple comparisons (respective *p*-values are given in the panels **B** and **D**).

Furthermore, femoral head slices treated under all conditions, including NaN_3_ pre-treatment, exhibit intense staining, again indicating that there is strong unspecific staining (Figure 4A). Nevertheless, the quantitative analysis revealed a significantly higher staining intensity in viable samples stained 3–6 h after explantation, compared to NaN_3_ pre-treated samples (Figure 4C versus 4D).

**Figure 5:**
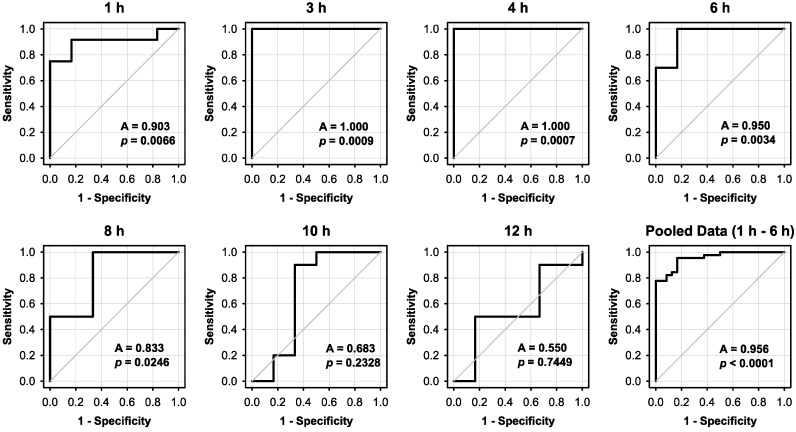
ROC-analysis of TTC-staining. ROC-analysis was performed with the data sets of native and TTC-stained bone slices obtained for each individual time point, which were shown in Figure 2. Time after explantation is given above the individual panels. Areas under the curve (A) and *p*-values are indicated within the panels.

The distinguishing power between remaining blood and viable, TTC-stained cells was analysed by receivers operating characteristics (ROC)-analysis, which is a widely used method for the evaluation and optimisation of analyses and tests. The ROC-curve provides a good visualisation of the dependence between sensitivity and fault rate (1-specificity). ROC-analyses were performed with the data sets of native and TTC-stained bone slices obtained for each individual time-point, which are illustrated in Figure 2. Despite the relatively low number of slices per time-point, the results confirm that TTC-stained viable sections treated up to six hours after explantation can well be distinguished from unstained sections: area under the curve greater 0.9 and *p* < 0.0066 for each individual time-point (Figure 5). Pooled data for stainings performed up to six hours post-explantation resulted in an area under the curve of 0.956 with *p* < 0.0001.

**Figure 6:**
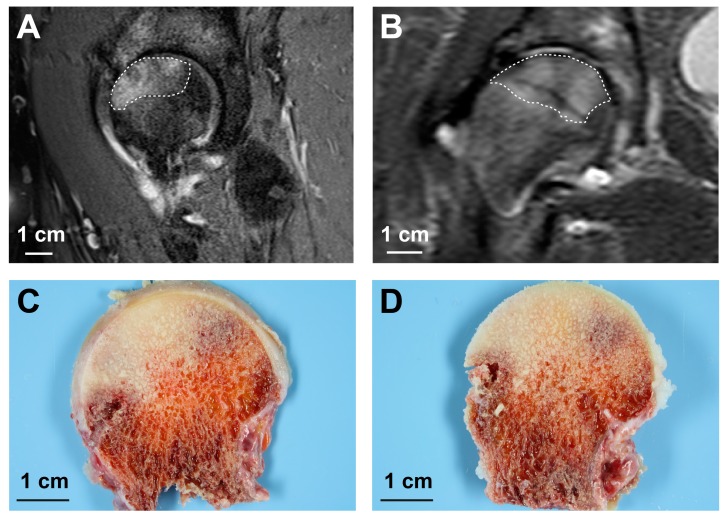
TTC-staining of bone slices from donors with femoral head necrosis. Hip MRI: (**A**) sagittal T1 fat saturated sequence from a 49-year-old female patient; and (**B**) coronal T1 STIR (Short Tau Inversion Recovery)-sequence from a 55-year-old male patient, both diagnosed with osteonecrosis of the right femoral head (dotted lines). The necrotic areas correspond with the unstained regions after TTC treatment of bone slices prepared from the femoral heads (**C**,**D**). Note that the angles of view differ slightly between MRI images and bone slices.

In an attempt to prove the ability of the staining procedure to detect non-viable bone areas, TTC-stained sections of femoral heads were compared with MRI images from two donors with femoral head necrosis. Indeed, the necrotic areas (as confirmed by MRT imaging) exhibit almost no coloration after staining with TTC (Figure 6).

## 3. Discussion

### 3.1. Potential Application for Vital Staining

This is the first description of the application of TCC-staining on human bone, using femoral heads as a model system. This simple procedure allows a macro-histological assessment of the vitality of a bone sample [4].

In everyday clinical routine, the question of the further operative treatment of fractures occurs fairly frequently. Oftentimes, fractures with joint involvement are not recognized in a timely manner and cannot be treated promptly because of this delay. The preservation of the joint is the chief objective of operative treatment—an endoprothetic replacement should only be a secondary option [5,6,7,8,9,10,11]. However, the viability of this approach is dependent on the vitality of the involved fracture fragments. The affected bone fragments of intra- or juxta-articular fractures are often threatened by posttraumatic lack of perfusion due to a rupture of afferent (nutrient supplying) vessels during the accident. Fracture healing (post-operative outcome), especially in the case of fractures of large joints, seems to be dependent on the time span between the accident and operative treatment [12,13]. A particular study shows that a six-hour boundary seems to exist [14]. This corresponds with our own results, which reveal that the cellular vitality of the bone decreases significantly after six hours—to a level similar to detection rates found in dead bone material.

Fracture healing and bone regeneration is a complex process that involves a combination of many biological factors (for review [15,16,17,18,19]. A vast variety of growth factors, peptides or hormones coordinate the activities of various cell types (e.g., osteoblasts and osteoclasts) via complex signalling networks [20,21,22,23,24,25]. In addition, mechanical stimulation [26], local inflammatory activity [27] or factors controlling osteoporosis [28] are involved in the process of fracture healing. The complexity and the intricate processes of signalling in bone formation during clinical fracture scenarios are not fully understood [26]. Importantly, no single and easily measurable parameter exists, which allows on-site prediction of fracture healing.

Since the vitality of the fracture fragments is essential for a positive healing process, an assessment of this factor would be indispensable in any effort to maximize the proportion of joint-preserving operations. An intraoperative evaluation of the vitality of the bone can only be indirect, e.g., blood flow from the drill channels, although this represents no absolute evidence for bone vitality. Similarly, intraoperative micro-perfusion imaging via laser Doppler does not allow conclusions on vitality [29,30]. Therefore, an instant intraoperative detection of the cellular vitality of the bone is hitherto not possible, since a real-time staining method that would allow a reliable assessment of cellular vitality is still not available. Unfortunately, the results of clinical laboratory testing are only available days (or even weeks) after sampling.

Evidence of the transferability of the TTC-staining method on the human bone would therefore enable an intraoperative assessment of a bone’s vitality. As a result, the further operative approach could be directly influenced, taking into account the operator’s thereby acquired knowledge of the bone vitality. The decision between osteosynthesis or joint replacement is, for example, critical in the case of older joint (or juxta-articular) fractures. To test for sensitivity and specificity of the TTC assay on bone samples, we used ROC analysis. This analytical tool confirmed the discriminatory power of the staining procedure for vital and non-vital bone. Comparison of MRI images and TTC-stained sections from patients with femoral head necrosis further confirmed the suitability of TTC-staining for the detection of vital bone. While MRI examinations are expensive and capacity-constrained procedures, the testing method described in this manuscript constitutes a simple method that is not only suitable, but also meets the economic and infrastructural demands of everyday clinical routine. Therefore, this method of vital staining could be used as a prospective predictive marker for the occurrence of posttraumatic avascular necrosis in further studies.

### 3.2. Technical Aspects of the Vital Staining 

The described staining method utilizes the conversion of TTC to a visible dye in vital cells via a mechanism that requires an intact respiratory chain and mitochondrial membrane potential. These are also the basis for the generation of sufficient levels of ATP in vital tissue. Non-vital, damaged tissue remains unstained because of its lack of intact mitochondria [1,2,3]. In this study, we have shown that TTC-staining indeed enables a visual, as well as an imaging-based, differentiation of viable and dead (NaN_3_-treated) bone [31]. Furthermore, stained viable bone can be differentiated from blood/erythrocyte contaminated bone. In the native group, the non-uniformity of the coloration of the cancellous bone is clearly observable. The reddening can ultimately be traced back to bleedings during the explantation and the preparation of the bone (slices). Furthermore, the extent of redness in the native group is significantly lower than in the TTC-stained group. Executing the staining assay always would include the comparison of the colour intensity of any given sample before and after staining, respectively. It can be assumed that images are necessary for documentation. Side by side inspection of these images should be sufficient for a decision concerning the viability of the stained bone fragment (image quantitation could be considered an add-on). Any increase in reddening after the staining procedure can only result from viable bone cells, as the red blood cells lack mitochondria.

In addition, the major information obtained by the staining method is only marginally affected by (minor amounts of) contaminating blood at all: the method identifies non-viable bone through absence of staining. In other words, it helps the operator to come to the conclusion that a defined bone area is definitively non-viable.

In an attempt to improve further visual differentiation of blood-contaminated bone and stained vital bone, we tested another tetrazolium-based staining procedure, which utilises MTT. MTT-staining is widely used for cell culture applications and has better staining properties—it is faster, more intense, and yields a blue-violet product [32]. This stain is also used to assess the viability of cultured osteoblasts [33]. However, our finding of severe background staining in bones, which is unrelated to the activity of viable cells, indicates that MTT is not suited for the application in bone tissue. Most likely, some component(s) of the extracellular bone mass causes this unspecific staining, which seems to be dependent on the subtle differences between the two stains.

Both results of our experimental study—the proven applicability of TTC and the unexpected findings that exclude the application of MTT—should be helpful in further comprehensive efforts to identify a faster stain with improved visibility in future studies.

Noteworthy, for a practical application a constant temperature of 37 °C has to be maintained during the staining procedure and the whole experimental setup. In prior pilot tests it became evident that the bone slices exhibited a reduced stainability at temperatures below 30 °C. Initial staining conditions found in the literature concerning application in heart and brain tissues, i.e., 5–30 min incubation time with 1–2% (*w*/*v*) TTC revealed almost no staining reaction [1,2,3]. Only an increased of the TTC-concentration to 3% yielded consistent staining after 20 min of incubations, which could not be intensified during longer incubations. We would recommend that anyone using this method should familiarize himself with the staining method (on a few bone fragments) beforehand.

TTC-staining could be performed intraoperatively. For this purpose, bone fragments could be obtained using hollow drills and could be stained immediately (the procedure from biopsy to result can be completed in 23 to 25 min). The restoration of stained bone fragments into the human body should be strictly avoided, as tetrazolium stains may be cytotoxic to some degree [34].

## 4. Materials and Methods

### 4.1. Patient Characteristics

After the local ethics committee’s approval of the prospective study, patients who were eligible to be included were informed in detail about the nature of the study according to the Declaration of Helsinki. All patients included in this study gave their informed consent.

Cases with primary and post-traumatic coxarthrosis, as well as osteoarthrosis caused by dysplasia, were included in the study—in these cases of planned unilateral total endoprothetic replacements, the explanted femoral heads were conserved for later TTC-staining. Exclusion criteria comprised prior operations in proximity to the hip joint, necrosis of the femoral head, intake of bone metabolism active medications, bone metabolic diseases and patients with immunomodulatory medications. In total, 37 patients (18 male/19 female) were included in the study: ages were 66.7 ± 10.3/63.2 ± 12.0 years and body mass indices were 27.1 ± 3.0/28.6 ± 5.7 kg·m^−2^ (means ± standard deviations). A total of 296 bone slices were prepared from the femoral heads, 281 of which have been used in this study.

### 4.2. Feasibility Studies with Bone Fragments

To test whether TTC-staining might be suitable for the assessment of bone vitality, a pilot-study that used bone fragments was conducted. For that purpose, cancellous bone from femoral heads was extracted with a Luer bone rongeur. The fragments were pooled and stored in phosphate buffered saline (PBS) overnight at 4 °C. On the next morning, 250–300 mg of the cancellous bone fragments were placed in individual wells of 48-well tissue-culture plates (Greiner Bio-One, Frickenhausen, Germany) and reheated to 37 °C in PBS. For these experiments the placement into the incubator was defined as time = 0. At indicated times, PBS was replaced by 1 mL of 3% (*w*/*v*) TTC (Sigma-Aldrich, Taufkirchen, Germany)-solution in PBS. After 20 min, the staining solution was discarded, and the cancellous bone fragments were washed three times with PBS. For negative controls, cancellous bone fragments were pre-incubated with the respiratory chain toxin sodium azide (NaN_3_, 0.05% *w*/*v*) for 20 min, and washed three times with PBS before staining [31]. To exclude the possibility that NaN_3_ simply inactivates the TTC-stain, the staining solution from NaN_3_-treated wells was used for the staining of untreated cancellous bone fragments thereafter. Thus it could be assured that lack of staining after NaN_3_-treatment was due to dead cells and not inactivated dye. The fragments were transferred into pre-weighed 1.5 mL reaction tubes to allow for an exact mass determination. Thereafter, 1 mL 2-propanol was added and the red reaction product of the TTC-staining was extracted at 37 °C and 750 rpm on a Thermomixer (Eppendorf, Hamburg, Germany). The extracts were transferred into new tubes and centrifuged for 5 min at 4 °C at 20,000× *g*. Three 200 µL replicates of the supernatants were transferred to 96-well plates and absorbances were measured at 492 nm using a microplate-spectrophotometer (SpectraMax M2; Molecular Devices, Sunnyvale, CA, USA). After the correction of absorbance values for blank absorbance, the means of the triplicate replicates were divided by the mass of the corresponding cancellous bone fragments. Results were expressed as µAU (absorbance units) per mg cancellous bone.

### 4.3. Preparation of Bone Slices and TTC-Staining Procedure

After removal, the femoral heads were sawn into 2.5 mm bone slices, using a custom made template and a customary bone saw (Aesculap, Tuttlingen, Germany) with an oscillating saw blade (Ulrich, Ulm, Germany). To remove bone dust, the slices were brushed and flushed with isotonic saline (Isotonische Kochsalzlösung^®^, Fresenius, Bad Homburg, Germany). Thereafter, the femoral head slices were incubated in isotonic saline at 37 °C until being used for staining. Bone slices were either left native without any treatment, treated solely with TTC, or pre-treated with NaN_3_ and subsequently stained with TTC. To test whether staining would reveal different results at different time points, individual slices were treated 1, 3, 4, 6, 8, 10, or 12 h after explantation, respectively. Treatment time is defined as the time after explantation, when the staining solution was applied. Because a single femoral head could not yield slices for all test conditions/times, assignment to the different test groups took place randomly.

For staining, the bone slices were incubated for 20 min in a 3% (*w*/*v*) solution of TTC in PBS at 37 °C under dark room conditions, with a single turning of the slices. Subsequently, images of the anterior and posterior sides of all bone slices were obtained under standardized conditions with an EOS 1000 D camera (Canon, Tokyo, Japan). Both sides of the bone slices were photographed, measured and analysed independently. The means were used for further analysis. In the case of bone slices where only one side showed a cancellous bone that was assessable, only this side was photographed and consequently evaluated.

For the NaN_3_-treatments, bone slices were incubated in a 0.1% (*w*/*v*) NaN_3_-solution at 37 °C for 20 min, and afterwards flushed with isotonic saline. Thereafter, the slices were stained with TTC in the manner described above and consequently photographed under the same standardized conditions.

Quantitative analysis of TTC-staining was performed with the FIJI software package [35]. Briefly, images (*n* = 4–12 per condition) were converted into an 8-bit format. Identical threshold (low: 80, high: 255) was applied to all images and a region of interest was selected to cover the cancellous bone, with the exception of areas of the femoral head that were damaged by the necessary drilling with a corkscrew extractor, as well as bone cysts. Mean intensity within the selection was measured.

### 4.4. MTT-Staining

The properties of the related 3-(4,5-Dimethylthiazolyl-2)-2,5-diphenyl-2H-tetrazoliumbromide (MTT, Thiazolyl blue)-stain suggested a faster staining and a better contrast in relation to remaining blood. MTT-staining and quantitation were essentially performed in the same manner as TTC-staining (as described above), with some slight modifications: the concentration of the staining solution was 0.5 mg MTT·mL^−1^; staining was performed for 10 min; and for spectrophotometric measurements the differences of absorbances obtained at 570 and 690 nm were used, respectively.

### 4.5. MRI-Imaging

In order to allow a comparison between an established method for the diagnosis of bone necrosis [36] and our own testing method, we exemplarily included two patients with bone necrosis initially diagnosed by MRI-imaging. MRI-images were generated on standard instruments.

### 4.6. Statistical Analysis

For descriptive statistics of normally distributed data sets mean ± standard error was used to summarize the integrated density of the different measurements. When at least one of the compared experimental groups lacked normality, box plots were used, where boxes represent 25th and 75th percentiles, respectively. Medians are indicated by a horizontal line. Whiskers indicate 10th and 90th percentiles, respectively. For intra- and inter-group comparison, analysis of variance (ANOVA) was utilized and all pairwise multiple comparisons were performed with the Holm-Sidak test, if all data sets were normally distributed. Otherwise, Kruskal-Wallis ANOVA on ranks (KWAR) with Dunn’s multiple comparison was utilized. In addition, receivers operating characteristics (ROC)-analysis was performed to compare selected groups. *p*-values of less than 0.05 were considered statistically significant.

## 5. Conclusions

TCC-staining is an easy method to assess cell activity and viability. Up to this point, this method has only been used under laboratory conditions to assess the vitality of the heart muscle and of cerebral nerve cells. We were able to show for the first time that TCC-staining is also functional in regards to the human bone. The described simple procedure could allow an intra operative decision whether the concerned bone appears vital or non-vital. However, development of a faster protocol for staining, i.e., by means of a catalysing agent, seems imperative for routine applications. For the first time, the operating surgeon has the possibility to make a decision about his further surgical approach based on a direct assessment of the vitality of the concerned bone. A potential application could be the intraoperative decision making whether a bone conserving or a bone replacing approach is appropriate in cases of older joint (or in joint-proximity) fractures. Furthermore, this intraoperative staining method could be used in the context of multi-fragmented fractures. Whether a fragment becomes a sequestrum, with its inherent potential for damage, or whether it reintegrates into the bone structure is primarily dependent on the vitality of the fragment.
